# Impact of an Asian Community-Based Cancer Rehabilitation Program on Health-Related Quality of Life

**DOI:** 10.3390/healthcare12222251

**Published:** 2024-11-11

**Authors:** Matthew Rong Jie Tay, Chin Jung Wong, Vijayalaxmi Chadachan

**Affiliations:** 1 Department of Rehabilitation Medicine, Tan Tock Seng Hospital, 11 Jalan Tan Tock Seng, Singapore 308433, Singapore; 2 Singapore Cancer Society Rehabilitation Center, National Cancer Center Singapore Building, Singapore 168583, Singapore; vijayalaxmi_chadachan@singaporecancersociety.org.sg; 3 Lee Kong Chian School of Medicine, Nanyang Technological University, 11 Mandalay Road, Singapore 308232, Singapore

**Keywords:** health-related quality of life, patient-centered outcomes, oncology, rehabilitation, exercise therapy, cancer survivors

## Abstract

Background/Objectives: Inpatient exercise-based rehabilitation has been shown to improve health-related quality of life (HRQOL) in cancer survivors. However, there is a lack of studies on the impact of community-based cancer rehabilitation programs on health-related quality of life, especially in Asian countries. Methods: This was a retrospective cohort study involving patients with cancer at an outpatient community-based rehabilitation center. There were 197 patients who were recruited and enrolled in a physician-led rehabilitation program which included physiotherapists, occupational therapists, nutritionists and exercise physiologists. Results: Most of the patients had a diagnosis of breast cancer (61.4%), while 76 (38.6%) had a diagnosis of other cancers. On initial assessment, we found a mean Distress Thermometer (DT) level of 3.37 (SD = 2.41) and a mean Functional Assessment of Cancer Therapy-General-7 Item Version (FACT-G7) score of 11.83 (SD = 4.01). On follow-up assessment after 3–6 months of rehabilitation, there was a significant reduction in mean DT level to 2.42 (SD = 2.25) and an improvement in mean FACT-G7 score to 13.09 (SD = 4.77). Multivariate regression analysis revealed that significant factors for improvement in FACT-G7 scores were age (*p* = 0.046) and number of exercise therapy sessions (*p* < 0.001). Conclusions: This study demonstrates the positive impact of a community-based cancer rehabilitation program on HRQOL among cancer patients.

## 1. Introduction

Increased cancer survival rates from advances in cancer detection and treatment have resulted in improved mortality for cancer survivors [1]. Despite more effective cancer therapeutics, the cancer itself and the treatment can adversely affect health-related quality of life (HRQOL). This has resulted in a growing number of cancer survivors who will experience debilitating complications including cancer-related fatigue, muscle atrophy, cardiopulmonary toxicity, lymphedema and psychosocial side effects, which can affect their HRQOL [2,3]. However, rehabilitation can play a key role in patients with a cancer diagnosis and who have received cancer therapies including surgery, radiation and chemotherapy. There is firm evidence that exercise-based multidisciplinary rehabilitation programs can improve fatigue and health-related quality of life in cancer patients [4], especially in inpatient rehabilitation settings [5,6,7,8].

However, community-based cancer rehabilitation programs pose challenges for healthcare providers and patients, including physical space restrictions and the need for professional guidance and supervision of programs [9,10,11,12]. While there has been a concerted effort to implement hospital-based outpatient cancer rehabilitation in Western countries with varying degrees of success [13,14,15], there has only recently been a focus on community-based cancer rehabilitation programs [16,17,18]. Such community interventions are not well studied in Asian populations, where barriers to structured outpatient rehabilitation programs exist, such as the paucity of healthcare professionals familiar with rehabilitation in cancer survivors, inadequate cancer-related health literacy among cancer survivors and a largely sedentary population [19,20,21,22]. Although there is high awareness of cancer survivorship care in Western countries, Asian patients may have poorer recognition of cancer-related impairments due to the lack of such services in these countries [23]. Additionally, there tends to be an emphasis on cancer surveillance in post-cancer follow-up, without recognizing the physical and psychosocial needs of cancer patients, in contrast to multidisciplinary models of survivorship care prevalent in North American and European countries [20].

Therefore, the objective of this study was to investigate the impact of an Asian community-based multidisciplinary cancer rehabilitation program on HRQOL.

## 2. Materials and Methods

This was a retrospective cohort study, which recruited Asian cancer patients who presented at a national community-based cancer rehabilitation center between 2018 to 2020. All cancer patients were referred by clinical specialists or primary care physicians from any local healthcare institution after they had completed their acute oncological treatment.

Inclusion criteria were adults ≥21 years old who had a cancer diagnosis and were enrolled in the rehabilitation program. The exclusion criteria were terminally ill patients, those undergoing active cancer treatment, and those with major psychiatric illness or functional limitations that made exercise unsafe or incomplete assessments. Patients with bone metastases were allowed to participate in the study. The clinical study was performed in accordance with the principles of the Declaration of Helsinki. Ethical approval was obtained from the local institutional review board, Agency for Integrated Care (2021-001).

### 2.1. Intervention

All patients first attended an initial assessment visit, where they would be assessed by a physiatrist. Depending on initial assessment, HRQOL, the level of psychological distress and function goals, they were then enrolled in a multidisciplinary physician-led rehabilitation program which included physiotherapists, occupational therapists, nutritionists and exercise physiologists, individualized to their needs. For example, a cancer patient who was assessed by the physiatrist to have adhesive capsulitis, deconditioning and loss of weight would be referred for physical therapy, nutritional therapy and exercise therapy sessions.

The program included physical therapy (e.g., manual therapy, balance retraining, joint mobility, physical modalities) and lymphedema management (e.g., lymphatic drainage, compression bandaging). Occupational therapy included Activity of Daily Living adaptations and fatigue management. Nutrition therapy was provided by a dietician, including lifestyle interventions and weight loss during and after rehabilitation. Physical therapy, occupational therapy and nutritional therapy were provided for 1 h each session. For example, patients with adhesive capsulitis undergoing a physical therapy session could have a heat or ice pack applied to relieve pain before commencing exercises. Patients then received joint mobilization and stretching exercises. Strengthening exercises can be added to maintain muscle strength, with isometric or static contraction exercises provided if pain was severe. Patients would then be prescribed a home exercise program. Patients with lymphedema may receive education on the care of the affected limb, may be taught lymphedema massage techniques and may receive compression bandaging if the lymphedema was significant. Patients with cancer-related fatigue undergoing an occupational therapy session may receive therapeutic exercise, interventions to improve Activity of Daily Living, durable medical equipment recommendations and may be taught energy conservation techniques.

For exercise therapy, patients met with a supervising exercise physiologist for an individualized exercise program according to each patient’s medical comorbidities, cancer diagnosis and stage, cancer treatment regimen and functional goals. They were then enrolled in an exercise therapy program involving supervised resistance and aerobic training up to twice weekly for 12 weeks, for a duration of 1h each time.

In addition, patients were encouraged to undertake home-based aerobic exercise sessions of walking, with the aim of accumulating a total of 150 min of moderate intensity exercise a week [9]. All patients received at least 1 session of physical, occupational, nutritional, or exercise therapy. All patients also received general counseling on general health behavior and on coping with cancer. Most patients were enrolled for a period of between 3 to 6 months of intervention.

### 2.2. Outcome Assessment

The primary outcome measure of this study was HRQOL, which was measured by the Functional Assessment of Cancer Therapy-General-7 Item Version (FACT-G7) [24], a modified version of the Functional Assessment of Cancer Therapy-General (FACT-G) questionnaire [25]. This is a rapid index of 7 high-priority FACT-G items which is used to evaluate symptom burden and HRQOL in cancer patients over time, of which 3 items are from the physical well-being subscale of the FACT-G (fatigue, pain and nausea), 1 item is from the emotional well-being subscale of the FACT-G (worry about condition worsening) and 3 items are from the functional well-being subscale (enjoyment of life, contentment with quality of life and sleep). The FACT-G7 has demonstrated good validity and reliability in the cancer population [26]. It also shows good internal consistency and concurrent validity in Chinese patients [27]. This rapid questionnaire takes only a few minutes to complete and requires little assistance [24]. The total score ranges from 0–28, with a score of 16 or lower indicating low HRQOL [28].

The other primary outcome measure was the Distress Thermometer (DT). A DT is a single-item self-reported measure of distress. Patients were asked to grade their distress in the past week on an 11-point visual analog scale ranging from 0 (no distress) to 10 (extreme distress) [29]. A DT score of ≥5 was used as a cutoff indicating a clinically significant level of distress [30,31]. The DT has been widely validated in Asian populations against longer and more burdensome tools like the Hospital Anxiety and Depression Scale, including patients in Korea [32], Taiwan [33], Japan [34], Indonesia [35], Malaysia [36] and Singapore [30].

Assessments were performed prior to enrolling in the rehabilitation program, and 3–6 months after intervention. Additional socio-demographic and clinical data were collected from patient records. The presence of any adverse events was also recorded.

### 2.3. Statistical Analysis

Descriptive statistics of demographic and clinical variables were presented as frequencies or mean ± SD depending on the variable.

Comparison of baseline and post-intervention HRQOL measures were performed using paired *t* tests or McNemar tests for continuous and categorical variables respectively.

A multivariable logistic regression model was used to determine which socio-clinical variables were associated with a post-intervention FACT-G7 or DT score above their pre-intervention values. These models investigated covariates including age, gender, ethnicity, education level, employment status, cancer type, cancer stage, treatment modalities (surgery, chemotherapy, radiotherapy, hormonal therapy) and number of therapy sessions (physical therapy, occupational therapy, nutrition therapy and exercise therapy).

All estimates were reported along with the 95% confidence interval (CI). Statistical analyses were performed using SPSS version 26.0 (IBM Corp., Armonk, NY, USA). All statistical tests were performed at a two-sided 5% significance level. There were no missing data.

## 3. Results

We recruited a total of 197 patients who enrolled in the rehabilitation program. There was no patient attrition during the study period. The mean age was 62.5 years, with 113 (57.4%) patients above or equal to the age of 65, and 84 (42.6%) patients below the age of 65. There were 121 patients (61.4%) with a diagnosis of breast cancer, while 76 (38.6%) had a diagnosis of other cancers. The other cancer diagnoses were 21 urological cancers (10.6%), 14 colorectal cancers (7.1%), 13 gynecological cancers (6.6%), 8 lung cancers (4.1%), 11 hematological cancers (5.6%), 4 head and neck cancers (2.0%), 3 brain cancers (1.5%) and 2 hepatobiliary cancers (1.0%). In the study cohort, 98 patients (49.7%) had no recurrence of cancer, while 34 patients (17.3%) had localized/regional cancer, and 65 patients (33.0%) had metastatic cancer. Patients received an average of 3.41 physical therapy, 2.49 occupational therapy, 1.45 nutritional therapy and 6.79 exercise therapy sessions. (Table 1).

The period between the initial and follow-up assessments ranged from 3 to 6 months, with a mean duration of 4.38 (±1.05) months. On initial assessment, DT level was a mean of 3.37 (±2.41), with 68 (34.5%) patients reporting a significant amount of distress, and FACT-G7 scores had a mean of 11.83 (±4.01), with 173 (87.8%) patients reporting a low HRQOL. On follow-up assessment, there was an improvement in FACT-G7 scores to a mean of 13.09 (±4.77), with only 47 (23.9%) patients reporting a low HRQOL (*p* < 0.005). Additionally, there was a significant reduction in DT level to 2.42 (±2.25), with 39 (19.8%) patients reporting significant distress (*p* < 0.001) (Table 2).

On multivariate analysis, significant associations with an improvement in baseline FACT-G7 scores were age ≤ 65 (OR = 2.09; 95% CI = 1.01–4.33; *p* = 0.046) and number of exercise therapy sessions (OR = 1.27; 95% CI = 1.16–1.39; *p* < 0.001) (Table 3).

Significant associations with an improvement in baseline DT scores were age ≤65 (OR = 2.81; 95% CI = 1.11–7.11; *p* = 0.029), no cancer recurrence (OR = 4.43;95% CI = 1.35–14.60; *p* = 0.014), localized/regional cancer (OR = 4.24; 95% CI = 1.04–17.28; *p* = 0.044), and number of exercise therapy sessions (OR = 1.57; CI = 1.38–1.80; *p* < 0.001) (Table 4). No adverse events were reported during the study period.

## 4. Discussion

The present study investigated the effects of a rehabilitation program on distress levels and health-related quality of life (HRQOL) in a cohort of cancer patients. The study findings highlight the significant improvements in HQOL in cancer patients, after undergoing a community-based multidisciplinary rehabilitation program for 3–6 months.

We found that a significant proportion of patients had presented with significant morbidity, with a majority (87.8%) reporting low FACT-G7 scores, and 68% reporting high levels of psychosocial distress (68%), even though most of the patients have completed acute cancer therapy and were expected to return to their former level of functioning in the community. This is similar to local studies which report high levels of psychological, physical and social problems in cancer patients in the community [37,38] and is indicative of the multifaceted challenges faced by individuals diagnosed with cancer. Additionally, most of the patients were diagnosed with breast cancer, aligning with global trends that highlight the prevalence of breast cancer among women, and the accompanying morbidity and psychological distress of cancer and oncological treatment [39,40]. This shows the clinical relevance of community-based rehabilitation for cancer survivors after acute cancer treatment, who require multidimensional interventions targeted at their physical and psychosocial needs.

The observed reduction in distress levels and the concurrent improvement in HRQOL emphasize the potential of exercise-based rehabilitation interventions in addressing the psychosocial and emotional well-being of cancer patients. We found that the number of exercise therapy sessions demonstrated a significant positive association with improvements in both distress levels and HRQOL. This highlights the importance and potential benefits of exercise therapy as an integral component of rehabilitation programs for cancer patients. Exercise rehabilitation has been shown to improve physical function, fatigue and depression, enhancing overall HRQOL [41]. A rehabilitation program based in the community where the majority of cancer survivors live and work is essential for survivorship care [42,43]. It also provides structure, supervision and social support that contributes to the adoption and adherence to a routine physical activity program [44]. Various contributors to the effectiveness of a community program include close integration of healthcare professionals to address common cancer-related morbidities (e.g., lymphedema, fatigue), camaraderie among cancer patients and having a dedicated cancer rehabilitation facility [45].

Multivariate analyses revealed various factors influencing the improvements in distress levels and HRQOL among the patients. Age emerged as a significant factor, with patients aged ≤65 exhibiting a greater likelihood of experiencing improvements in both distress levels and HRQOL. This possibly reflects the lower physical functional capacity and higher incidence of frailty in older patients prior to the rehabilitation program [46]. This finding underscores the potential role of age as a determinant of the effectiveness of rehabilitation interventions. Healthcare and exercise professionals should consider the patient’s physiological reserve at the time of screening and during rehabilitation, and tailor various exercise interventions to target different age groups and functional capabilities.

The absence of cancer recurrence was strongly associated with improvements in distress levels, suggesting that these patients may experience better psychological well-being and lower levels of distress. Similarly, patients with localized or regional cancer exhibited significant improvements in distress levels, compared to patients with metastatic disease, emphasizing the potential impact of cancer stage on HRQOL outcomes. These findings suggest that the physical and psychological burdens associated with tumor recurrence or metastatic disease may hamper or slow the recovery in HRQOL [47]. However, it should be noted that ongoing oncological treatment and metastatic disease (including bone metastases) are not contraindications to exercise rehabilitation, in the setting of appropriate exercise prescription [48,49]. Despite these negative prognostic factors, patients still reported an overall HRQOL improvement after rehabilitation, highlighting the benefits of rehabilitation even in patients with cancer recurrence or metastatic disease. The absence of reported adverse events further highlights the safety and feasibility of implementing rehabilitation programs for this category of patients.

Several limitations warrant consideration. First, we did not measure specific tests of physical function at baseline and post-intervention, nor did we obtain data on the specific exercises performed. Exercise prescription was left to the discretion of the healthcare professional based on individualized assessment. Second, the relatively short follow-up duration of 3–6 months may not capture the long-term effects of the rehabilitation program on distress levels and HRQOL. Third, we are also unable to adjust for additional rehabilitative treatment received by patients outside the community-based program. Fourth, although patients did report high levels of distress, our rehabilitation program did not have the resources for dedicated psychological health sessions, although general psychoeducation was provided as part of holistic care. Lastly, the lack of a control group limits the ability to draw definitive causal inferences regarding the effectiveness of the rehabilitation program, and future studies with randomized trial designs are required to provide a more balanced interpretation of our findings.

## 5. Conclusions

In conclusion, this study reports the positive impact of a community-based cancer rehabilitation program on HRQOL among cancer patients. Our findings also underscore the critical need for tailored rehabilitation and comprehensive interventions that consider the diverse needs of patients, including those of different age groups and cancer stages. Future studies with extended follow-up periods are warranted to validate the observed associations, measure the psychosocial impact and value effectiveness of such programs in detail and investigate barriers to adherence to exercise programs among cancer patients.

## Figures and Tables

**Table 1:** Clinical characteristics of the study population.

Variable	N = 197
Age, n (%)	62.5 (10.6)
- ≤65	113 (57.4)
- >65	84 (42.6)
Gender, n (%)	
- Male	37 (18.8)
- Female	160 (81.2)
Ethnicity, n (%)	
- Chinese	181 (91.9)
- Malay	12 (6.1)
- Indian	4 (2.0)
Education level, n (%)	
- 0–6 years	5 (2.5)
- 7–12 years	51 (25.9)
- >12 years	141 (71.6)
Employment status, n (%)	
- Unemployed	115 (58.4)
- Partial/full time employment	82 (41.6)
Cancer type, n (%)	
- Breast	121 (61.4)
- Other	76 (38.6)
Cancer stage, n (%)	
- No recurrence	98 (49.7)
- Localized/regional	34 (17.3)
- Metastatic	65 (33.0)
ECOG Performance Status, n (%)	
- 0–1	171 (86.8)
- 2 or more	26 (13.2)
Cancer treatment modalities, n (%)	
Surgery	129 (65.5)
Chemotherapy	175 (88.8)
Radiotherapy	178 (90.4)
Hormonal therapy	99 (50.3)
Therapy sessions, n (%)	
- Physical therapy	3.41 (1.76)
- Occupational therapy	2.49 (1.25)
- Nutritional therapy	1.45 (0.83)
- Exercise therapy	6.79 (4.86)

ECOG: Eastern Co-operative Oncology Group.

**Table 2:** Outcome measures of study population.

	Baseline	Follow-Up	*p* Value
FACT-G7 total scores, mean (±SD)	11.83 (±4.01)	13.09 (±4.77)	<0.001
Low FACT-G7 (≤16), n (%)	173 (87.8)	47 (23.9)	<0.001
Distress thermometer, mean (±SD)	3.37 (±2.41)	2.43 (±2.25)	<0.001
Distress thermometer score ≥5, n (%)	68 (34.5)	39 (19.8)	<0.001

FACT-G7: Functional Assessment of Cancer Therapy-General-7 Item Version.

**Table 3:** Multivariate analysis of variables associated with improvement in baseline FACT-G7 scores.

Variable	Multivariate Analysis	
	Odds Ratio (95% Confidence Interval)	*p* Value
Age ≤ 65	2.09 (1.01–4.33)	0.046
Gender (Female)	0.602 (0.156–2.31)	0.460
Ethnicity		
- Chinese	Reference	
- Malay	1.31 (0.231–7.43)	0.759
- Indian	0.351 (0.029–4.27)	0.412
Education level		
- 0–6 years	Reference	
- 7–12 years	0.465 (0.042–5.09)	0.531
- >12 years	0.397 (0.040–3.97)	0.432
Employment status (Partial/full time employment)	1.62 (0.761–3.45)	0.211
Cancer type (Breast)	2.08 (0.647–6.71)	0.219
Cancer stage		
- No recurrence	1.08 (0.428–2.72)	0.873
- Localized/regional	0.480 (0.149–1.55)	0.219
- Metastatic	Reference	
ECOG Performance Status (0–1)	2.11 (0.731–6.09)	0.168
Surgery	1.33 (0.590–3.02)	0.489
Chemotherapy	0.330 (0.088–1.25)	0.102
Radiotherapy	0.713 (0.165–3.08)	0.651
Hormonal therapy	1.16 (0.398–3.38)	0.786
Number of physical therapy sessions	1.26 (0.987–1.60)	0.063
Number of occupational therapy sessions	0.769 (0.556–1.06)	0.113
Number of nutritional therapy sessions	0.816 (0.509–1.31)	0.399
Number of exercise therapy sessions	1.27 (1.16–1.39)	<0.001

FACT-G7: Functional Assessment of Cancer Therapy-General-7 Item Version.

**Table 4:** Multivariate analysis of variables associated with improvement in distress thermometer scores.

Variable	Multivariate Analysis	
	Odds Ratio (95% Confidence Interval)	*p* Value
Age ≤65	2.81 (1.11–7.11)	0.029
Gender (Male)	0.277 (0.52–1.47)	0.131
Ethnicity		
- Chinese	Reference	
- Malay	1.21 (0.32–2.45)	0.210
- Indian	0.643 (0.036–11.42)	0.764
Education level		
- 0–6 years	Reference	
- 7–12 years	7.07 (0.278–19.78)	0.236
- >12 years	11.02 (0.504–23.75)	0.127
Employment status (Partial/full time employment)	0.752 (0.296–1.91)	0.549
Cancer type (Breast)	1.17 (0.230–5.97)	0.850
Cancer stage		
- No recurrence	4.43 (1.35–14.60)	0.014
- Localized/regional	4.24 (1.04–17.28)	0.044
- Metastatic	Reference	
ECOG Performance Status (0–1)	0.978 (0.259–3.70)	0.974
Surgery	1.48 (0.520–4.23)	0.461
Chemotherapy	0.867 (0.147–5.12)	0.875
Radiotherapy	0.733 (0.128–4.22)	0.728
Hormonal therapy	0.580 (0.137–2.46)	0.460
Number of physical therapy sessions	1.27 (0.934–1.72)	0.128
Number of occupational therapy sessions	1.37 (0.906–2.06)	0.136
Number of nutritional therapy sessions	0.583 (0.299–1.14)	0.113
Number of exercise therapy sessions	1.57 (1.38–1.80)	<0.001

## Data Availability

The data presented in this study are available on request from the corresponding author.
